# CRISPR–Cas9-based genetic engineering for crop improvement under drought stress

**DOI:** 10.1080/21655979.2021.1969831

**Published:** 2021-09-10

**Authors:** Abdul Sami, Zhao Xue, Saheera Tazein, Ayesha Arshad, Zong He Zhu, Ya Ping Chen, Yue Hong, Xiao Tian Zhu, Ke Jin Zhou

**Affiliations:** a Rapeseed Cultivation and Breeding Lab, Anhui Agricultural University, Hefei, China; b Pgrl CABB, University of Agriculture Faisalabad, Faisalabad, Pakistan; c Plant Physiology Lab, Quaid I Azam University, Islamabad, Pakistan

**Keywords:** Drought, CRISPR–Cas9, plant productivity, ABA regulation, ethylene, genome editing

## Abstract

In several parts of the world, the prevalence and severity of drought are predicted to increase, creating considerable pressure on global agricultural yield. Among all abiotic stresses, drought is anticipated to produce the most substantial impact on soil biota and plants, along with complex environmental impacts on other ecological systems. Being sessile, plants tend to be the least resilient to drought-induced osmotic stress, which reduces nutrient accessibility due to soil heterogeneity and limits nutrient access to the root system. Drought tolerance is a complex quantitative trait regulated by multiple genes, and it is one of the most challenging characteristics to study and classify. Fortunately, the clustered regularly interspaced short palindromic repeat (CRISPR) technology has paved the way as a new frontier in crop improvement, thereby revolutionizing plant breeding. The application of CRISPER systems has proven groundbreaking across numerous biological fields, particularly in biomedicine and agriculture. The present review highlights the principle and optimization of CRISPR systems and their implementation for crop improvement, particularly in terms of drought tolerance, yield, and domestication. Furthermore, we address the ways in which innovative genome editing tools can help recognize and modify novel genes coffering drought tolerance. We anticipate the establishment of effective strategies of crop yield improvement in water-limited regions through collaborative efforts in the near future.

## Introduction

In 2017, at least 3% of the world’s land area was affected by extreme drought [1]. Approximately a fifth of the world’s population does not have sufficient food to survive normally, and nearly a billion people are hungry every year [1,2]. Drought decreases agricultural productivity, contributing to food shortage, and it is therefore one of the major causes of undernourishment and hunger [2]. In the mid-twentieth century, drought was the key reason of the scarcity of world grain production relative to demand, causing the food security crisis [3]. Farmers suffer from economic losses when crops are damaged by drought. Ranchers must spend additional money on food and water for their livestock. Business that relies on agriculture, such as companies producing tractors and food, experience losses when drought threatens crops or livestock. Severe drought reduces crop yield and production because of the scarcity of water and soil moisture to support plant growth. Under drought conditions, farmers reduce the area under crop cultivation and only plant drought-tolerant crops [4].

## Impact of climate extremes on crop production

Agricultural food production is directly affected by climatic factors, such as rainfall and temperature. These factors regulate crop development and health, crop yield over time, and annual crop yield [5]. Environmental extremes are estimated to become more frequent due to climate change, which can adversely affect crop yield [7]. While the impacts of climate change on agriculture have been reported at various regional scales, adaptive improvements to advance cropping practices for mitigating the effects of drought on plant yield have never been explored [7]. In addition, the impacts of weather extremes on the yield and growth of rainfed and irrigated plants warrant extensive research, given that groundwater, a vital resource for irrigation during drought, is slowly depleting. Severe drought decreases plant yield because less water and soil moisture are accessible for plant development. Under drought conditions, farmers deliberately reduce area under crop cultivation, and only plant drought-tolerant plants. Nevertheless, the spatiotemporal variability of the effects of drought on yield must be considered to mitigate and minimize possible negative impact on plant production [8].

## Negative effects of drought stress on plant activity

**Figure 1.**
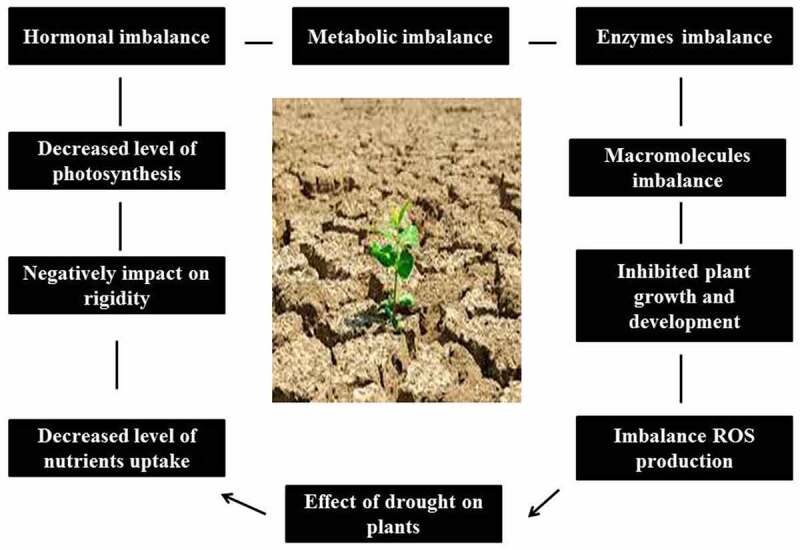
Drought stress significantly suppresses plant growth and development

Water scarcity elicits various crop responses at the physiological, molecular, biochemical, and morphological levels, eventually interrupting crop productively [9]. As shown in Figure 1, drought stress reduces plant production at various stages of growth. Indeed, reduced water supply decreases the germination and growth rate of crops [10]. During plant growth, drought affects plant water–interactions, which in turn disrupt the entire metabolism (at the molecular and physiological level), depending on the magnitude and duration of stress [11,12]. In addition, water scarcity alters plant processes, like suppression of photosynthesis [13,14] and reduction of crop productivity [15,16] are the major consequences. Under water deficit, indirect or direct oxidative stress serves as one of the key drivers of plant response, resulting in the alteration of biochemical machinery and destruction of cell membrane integrity, which further lead to severe metabolic complaints ultimately affecting plant function [17,18].

## Drought stress as a limiting factor for plant development

Drought is known as a limiting factor for several aspects of plant production. Germination, coleoptile length, and plant health are the key aspects of crop development [19]. Germination is a crucial step during development, and it is susceptible to drought. Notable changes in the seed germination of several crops, including wheat, sorghum, and maize, have been reported [20,21]. The noticeable signs of plants subject to drought stress at the early stages of vegetative growth include the disturbance of flowering, reduction in plant height, and wilting of leaves [22]. Drought often limits nutrient absorption of crops because of insufficient soil humidity, thereby suppressing stem development. In *Lathyrus sativus*, shoot length was reduced under water scarcity [23]. As a limiting factor, drought affects a wide spectrum of plant physiological processes, including growth and metabolism. Timing, duration, degree, and rate of development are critical factors taken into account during the selection of drought-tolerant species. Further, drought adversely affects several plant biological processes, from the embryonic to reproductive and maturation stages.

Plant drought tolerance mechanisms involve multiple biological processes at the level of cells, organs, and whole plant, which are activated at different plant developmental stages. For instance, plants reduce water loss through effective stomatal conductance and increase water uptake and transfer through the development of a highly efficient and deep root system (Figure 1). Furthermore, application of crop development regulators; preservation of membrane integrity; and use of appropriate plant genotypes, antioxidants, compatible solutes, stress-related proteins, and aquaporins support the development of drought-tolerant crops [24]. Moreover, plants with improved water use efficiency and enhanced antioxidant apparatus as well as those that can produce major osmolites and secondary metabolites are suitable as material to develop drought-tolerant crops. In addition, under water-limited conditions, compounds that can enhance drought tolerance of plants can be exogenously applied. Biotechnological approaches to produce drought-tolerant transgenic plants should also be considered, although their validity cannot precede the current field trials.

## Molecular mechanisms underlying plant drought tolerance

Improving agronomic traits that offer plant tolerance/resistance to abiotic and biotic stresses to enhance their economic worth has long been a global concern. Knowledge of global warming and climate change underlines the need to incorporate some practical and safe strategies. In some countries, the sustainability of crop production during drought is a significant problem. Drought intensity varies both temporally and spatially. Plants have evolved sophisticated responses and developed numerous physiological and morphological strategies to withstand stress. Specifically, plants face drought with differing degrees of adaptation, prevention, and escape [4,21]. The exploitation of genetic traits that improve drought response while retaining high yield remains crucial for plant management. Transgenic and conventional breeding methods could enhance the drought tolerance of wheat, soyabean, rice, and maize.

In the past, conventional breeding was the most productive way of growing plants, which encouraged their development in water-limited environments. However, these methods are labor-intestine, time-consuming, and expensive. Under stress conditions, molecular markers have played a pivotal role in characterizing the large variability in plant genetics [19]. In various crops, numerous quantitative trait loci (QTLs) conferring drought tolerance have been identified [25,26]. However, the accuracy and reliability of QTL identification remain problematic. In this light, genetic engineering has proven very successful in improving crops against abiotic and biotic stresses [27].

Innovative technologies that can expand the stress responses of plants and make them environment-ready are warranted. The introduction of genome editing tools has realized significant advances in plant breeding. Genome editing tools use sequence-definite nucleases to introduce specific known variations in the genome [28]. The CRISPR–Cas system of genome editing has been extensively acknowledged for its adaptability and ease of operation. This strategy uses a single guide RNA and complex Cas endonuclease that changes along the DNA strand to produce a double-stranded DNA breaks. Subsequently, these breaks are repaired by endogenic cell mending, leading to the expansion of novel mutations [29,30]. The CRISPR–Cas system has been professionally used for achieving resistance to multiple stresses, including heavy metals, salinity, drought, and submergence [31,32]. The present review emphases on the application of the CRISPR–Cas9 system to achieve drought tolerance in plants and discusses the potential of this technology in the expansion of drought-tolerant plant varieties.

Inclusive molecular studies have interpreted cellular pathways regulating plant response to drought [33,34]. Abscisic acid (ABA) plays crucial roles in regulating plant response to drought by controlling stomatal closure and gene expression to limit water loss via transpiration [11]. Basic leucine zipper (bZIP) transcription factor, called ABA-responsive element (ABRE)-binding proteins is essential for ABA signaling [33]. In soybean, rice, and *Arabidopsis, AREB1* over-expression (*ABF2*) improved drought tolerance, while *AREB1* loss-of-function increased drought susceptibility [35–39]. Furthermore, under drought, *AREB1* controls a broad range of genes downstream of the ABA signaling pathway [39] and is involved in ABA-mediated antioxidant signaling, ABA biosynthesis, and osmotic stress response. Therefore, *AREB1* is an attractive candidate to enhance plant response to drought [36,40–42].

Accessibility to genome sequences of many plants and advances in genome editing methodologies have opened new avenues of breeding for several desirable traits. Developments in genome editing tools, such as transcription activator-like effector nucleases (TALENs) and zinc-finger nucleases (ZFNs), have allowed molecular biologists to more specifically target any gene of interest. Nevertheless, these approaches are costly and laborious, since they need complex phases involving protein engineering. Unlike first-generation genome editing approaches, the CRISPR–Cas9 system requires easy operation and simple cloning procedures. The same Cas9 is tentatively available for use with various guide RNAs, targeting several sites in the genome. Following proof-of-concept demonstrations using a preliminary CRISPR–Cas9 unit in plants, numerous types of Cas9 cassettes (StCas9, SaCas9, and NmCas9) have been introduced to enhance target accuracy and reduce off-target cleavage. Furthermore, the availability of Cas9 enzymes from other bacteria has increased the accuracy and efficiency of gene editing [43,44]. The present review outlines the options open to agro-biotechnologists for crop enhancement using the established Cas9-based genome editing techniques. Cas9 enzymes have been used to enhance abiotic and biotic stress tolerance/resistance [27,45]. Implementation of these strategies is expected to produce non-genetically modified (non-GMO) plants with the objective phenotype, which may improve yield under abiotic and biotic stresses [21].

## CRISPR-based targeted genome editing for drought tolerance

**Figure 2.**
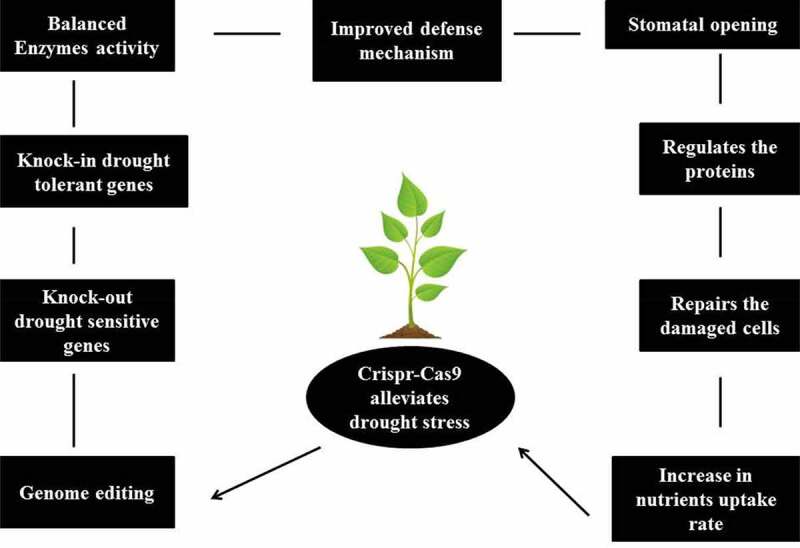
CRISPR–Cas9 alleviates drought stress and promotes plant growth and development

Various abiotic stresses substantially reduce crop yield by suppressing plant growth and reducing plant productivity [46]. Due to the dynamic nature of drought stress, genomic adaptation has previously been shown to be the sole approach to achieve drought tolerance. Overexpression of many genes and transcription factors related to drought signaling promote the aggregation of signaling molecules and metabolites and improve crop drought tolerance [47]. Meanwhile, the expressions of sensitive (S) genes increase plant drought susceptibility via hormonal imbalance, low antioxidant activity, and reactive oxygen species (ROS) generation. Stress-related ring finger protein 1 (OsSRFP1), drought-induced SINA protein 1 (OsDIS1), and dry- and salt-tolerant protein 1 (OsDST) are negative regulators of drought tolerance, whose silencing increased antioxidant enzyme levels, reduced H_2_O_2_ concentrations, and enhanced drought tolerance in rice [47,48]. Natural drought tolerance can be realized via genome editing to target drought-sensitive genes or negative regulators of abiotic stress response. As the first proof-of-concept study, the CRISPR–Cas9 system was used to introduce novel alleles in *Arabidopsis* OPEN STOMATA 2 (OST2)-encoding gene – a key plasma membrane H^+^ ATPase crucial for stomatal function [11]. Plasma membrane H^+^ ATPases are involved in the generation of proton gradient to initiate stomatal conductance [42]. In the presence of dehydration, ABA binds to the C-terminus of the proton pump, suppressing H^+^ ATPase function and inducing stomatal closure. Importantly, two significant mutations at the ost2 locus obliterate stomatal response to ABA, contributing to the constitutive functioning of proton pumps and induction of necrotic lesions [42]. Using an efficient CRISPR–Cas framework combining Cas9 and a truncated sgRNA (tru-sgRNA), mutations in transgenic crops could be identified with extraordinary efficacy (>32%) and without off-target alteration. Assessment of stomatal response under ABA-arbitrated conditions showed that compared with the wild-type, the *ost2*-CRISPR mutant exhibited a substantially higher rate of stomatal closure coupled with a lower rate of transcriptional water loss (Figure 2, Table 1). Therefore, CRISPR–Cas9-mediated mutations at the OST2 locus enhanced drought tolerance by improving stomatal response. Most recently, the CRISPR–Cas9 system was used to produce mutant lines of the non-expresser tomato of pathogenesis-related 1 (*NPR1*) to validate the role of this gene in drought tolerance [49]. Although *NPR1* is a key stimulator of the plant defense system, its function in abiotic stress is negligible. However, in drought-responsive apple plants, *MdNPR1* downregulation has been documented [50]. In rice, *AtNPR1* overexpression resulted in a hypersensitive response to drought stress (Li et al. 51). In wild tomato, CRISPR–Cas9-mediated loss-of-function *s1npr1* mutants exhibited weaker drought tolerance, higher stomatal opening rate, higher malondialdehyde (MDA) content, greater electrolytic leakage, and higher H_2_O_2_ levels, and lower antioxidant enzyme levels. Moreover, down regulation of drought-responsive genes, including *SIDREB, SIDHN*, and *SIGST*, further confirmed the drought susceptibility of *s1npr1* mutants (Table 1). Consequently, in tomatoes and other crops, *S1NPR1* plays critical roles in regulating drought response, and multiple *SlNPR1* variants can be produced using genome editing to confer broad-spectrum drought tolerance [49].

**Table 1.** Application of the CRISPR-based genome editing approach in plants for improvement of drought stress tolerance

Species	Target gene/site	Transformation method/Strategy	Target trait/Improved trait	Reference
*Arabidopsis*	Open stomata 2 (OST2)	Agrobacterium-mediated transformation	Drought stress tolerance by altering stomatal closing	[52]
*Arabidopsis*	miR169a	Agrobacterium-mediated transformation/ dual-sgRNA/Cas9-mediated targeted deletion to create null mutations	Targeting sensitive gene replacement by HDR	[53]
Maize	Auxin-regulated gene involved in organ size [ARGOS]	Biolistic mediated transformation/ CRISPR/Cas9-mediated DNA repair in untranslated regions of target genes to produce over expression	Overexpression of ARGOS8 to reduce ethylene sensitivity to enhance flowering/ increase grain yield under drought stress	[32]
*Arabidopsis*	Arabidopsis thaliana vacuolar H+- pyrophosphatase [AVP1]	Agrobacterium-mediated transformation/CRISPR/Cas9 activation system to produce overexpression of target gene	Enhanced number of leaves and leaf area	[54]
*Arabidopsis*	ABA-responsive element- binding protein 1 (AREB1)	Agrobacterium-mediated transformation/CRISPR activation system to enhance the expression of target gene	Increased chlorophyll cornetts and faster stomatal opening	[55]
Tomato (*Solanum lycopersicum*)	Mitogen-activated protein kinases 3	*A. tumeficiens/CRISPR cas9 system to knockout expression of Slmpak3 expression*	Protecting cell membrane from oxidative damage	[70]
Rice	SNF 1-related protein kinase 2	A. tumeficiens/CRISPR generated mutation in targeted genes	Inducing compatible solutes and decrease damage by ROS	[72]
*Arabidopsis*, Poplar	*PtoMYB216*	CRISPR Cas9 generated mutation of targeted gene	Regulates lignin deposition leading to flexible and collapsed xylem during wood formation	[56]
*Arabidopsis*	*UGT79B2, UGT79B3*	CRISPR generated mutations	Modulating anthocyanin accumulation	[57]
Cassava	*MeKUPs*	CRISPR generated analysis of KUP genes	Maintaining osmotic balance	[58]
Cassava	*MeMAPKK*	Activates MAPKK genes	Tissue development	[59]
Cotton	*GhPIN1-3* *GhPIN2*	CRISPR based gene targeting to control auxin distribution	Controls the cell growth and development	[60]
Cotton	*GhRDL1*	Characterizing promoter of a dehydration-responsive gene	GUS activity in trichomes also expression was observed in leaves, stems and floral tissues	[61]
Sugarcane	*ScNsLTP*	Targeting soluble proteins/nonspecific lipid transfer protein	Catalyzing phospholipids response	[62]
Wheat	*TaDREB2* *TaERF3*	CRISPR Cas9 genome editing in wheat protoplast for targeted genes manipulation	Maintained the expression of wheat dehydration responsive element binding protein 2 (TaDREB2) and wheat ethylene responsive factor 3 (TaERF3)	[63]
Papaya	*CpDreb2*	CRISPR generated gene disruption	Overexpression of targeted gene responsible for transmitting signals under water stress	[64]

## CRISPER–Cas9 and ABA regulation

Extensive molecular studies have revealed that ABA acts as the main drought response element in plants by affecting stomatal closure to inhibit water loss and regulating stress-related gene expression [11]. The bZIP unit (AREBs/ABFs) and binding domain (ABRE) of transcription factors, called ABA-sensitive factors, are the key components of ABA signaling [50]. *AREB1* upregulation improved drought tolerance, while *AREB1* knockout increased drought susceptibility. *AREB1* regulates the expression of a wide range of genes throughout the ABA signaling pathway and serves as a key element of osmotic stress response, antioxidant signaling, and ABA biosynthesis [14,65]. Therefore, *AREB1* may be used as an important target for improving plant drought tolerance.

In *Arabidopsis*, a modified CRISPR–Cas9 framework integrating sgRNA, catalytic domain of histone acetyltransferase (HAT) enzyme, and dead Cas9 (dCas9) was used to access the promoter region of *AREB1* [66]. The binding of *Arabidopsis* HAT catalytic domain led to acetylation of the central histone, enhancing the sensitivity of the *AREB1* promoter region to the transcriptional region. Physiological and molecular analyses of mutants showed higher *AREB1* expression, stomatal opening rate, and chlorophyll level under drought. In addition, *AREB1* activated *RD29A* expression under water-scarce conditions. The transgenic CRISPR lines exhibited improved survival rate under drought stress. Collectively, these results indicate that the CRISPR–Cas system can be successfully used to induce epigenetic alterations for enhancing plant drought tolerance via the positive control of drought-responsive genes.

SNF1-associated protein kinase 2 (SnRK2) a plant-specific protein kinase family – serves as the central stimulator of ABA-dependent hyperosmotic stress response and signaling [13]. SnRK2 members are commonly involved in seed germination, hyper-osmotic stress response, ABA-mediated stomatal closure, ABA signaling, drought tolerance, and seedling development [67]. In fact, in *Arabidopsis, AtSnRK2*.8 exhibited a typical stress regulatory network to positively control of drought tolerance via the upregulation of stress-responsive genes [68]. Despite the lack of significant differences in stomatal injury and survival between the wild type and *snrk2*.8 mutant, microarray analysis revealed that *SnRK2* regulated the AREB–ABF axis and its targets. Similarly, in rice, members of subclass I and III of the SnRK2 family improved plant growth and abiotic stress response [67].

## Effects of CRISPR on plant productivity

The noticeable signs of plants subject to drought at the early vegetative stages are reductio in plant height, wilting of leaves, and disruption of flowers and buds [69]. Severe drought often inhibits nutrient absorption by plants. In tomato, the CRISPR–Cas9 method was used to suppress mitogen-activated tomato protein kinase 3 (*slmapk3*) for elucidating the regulatory cascade underlying *SlMAPK3*-mediated drought tolerance (Figure 2, Table 1). Under drought conditions, the *slmapk3* mutant lines showed more severe stem curling and leaf wilting than the wild type lines [70]. Moreover, the mutant plants showed substantially higher H_2_O_2_, proline, and MDA levels than the wildtype plants, suggesting that the mutant lines experienced more severe oxidative stress and membrane injury under drought. These findings in tomato highlight the role of *SlMAPK3* in drought tolerance, offering insights into the *SlMAPK3*-regulated mechanism of drought tolerance [70]. Further research involving genetic engineering to augment *SIMAPK3* expression in tomato can improve yield and tolerance under drought conditions [71].

Similarly, under heat stress, CRISPR–Cas9-mediated knockout the tomato SLAGAMOUS-like 6 (*SlAGL6*) induced the formation of parthenocarpic fruits. The CRISPR–Cas9 system was used to produce the loss-of-function mutant of stress/ABA-activated protein kinase 2 (SAPK2), the key mediator of ABA signaling, in rice. This *SAPK2* rice mutant was more susceptible to oxidative and drought stresses than the wild type, suggesting that *SAPK2* is essential for drought tolerance in rice and can serve as a candidate gene for future crop development [72]. Similarly, in maize, the transcription standard of *ARGOS8-v2* and *ARGOS8-v1* was considerably higher than that of the wild type, and the *ARGOS8* variant showed substantially improved grain production under drought conditions and zero yield loss under normal growth situations [32].

At DuPont Pioneer, [32], documented that the maize variants modified using CRISPR were more drought tolerant. The authors also demonstrated that the CRISPR–Cas9-mediated ARGOS8 variants showed enhanced grain production under field conditions during the dry season. These findings indicate that the CRISPR–Cas9 method can be successfully and effectively used to induce novel allelic modifications for developing drought-tolerant crop varieties. Consistently, a potent CRISPR–Cas9 framework using tru-gRNAs and Cas9 driven by the tissue-specific promoter AtEF1 could successfully induce mutations of abiotic stress-sensitive genes (OST2/AHA1) with no off-target consequences [2,11]. In *Arabidopsis*, the novel OST2/AHA1 mutant alleles were created by streamlining the CRISPR–Cas9 system with intense stomatal responses. These findings paved the way for the application of CRISPR–Cas9-mediated genetic engineering to improve crop production and multigenic stress resistance [71].

## Identification of negative regulators of stress tolerance

Given the dynamic nature of drought conditions, successful use of genome editing to induce drought tolerance has previously been demonstrated. Overexpression of specific transcription factors and genes involved in drought stress signaling promotes the aggregation of signaling molecules and metabolites and improves drought tolerance of crops [47,73].

The binding of the *Arabidopsis* HAT catalytic domain triggered acetylation of the central histone, thereby enhancing the sensitivity of the *AREB1* promoter region to the transcriptional region. Physiological and molecular analyses of mutants revealed upregulation of the AREB1- and AREB1-regulated *RD29A* genes, rapid opening of stomata, and increase in chlorophyll concentration under water scarcity. The transgenic CRISPR lines exhibited improved survival under drought conditions. The drought tolerance gene *OsDREB* has been proposed as a target for the CRISPR–Cas9 system in rice [74]. Taken together, these reports indicate that the CRISPR–Cas method can successfully induce epigenetic alterations to enhance drought tolerance via the positive control of drought-responsive genes.

## Effects of CRISPR–Cas9 on ethylene responsive factors (ERFs) for drought stress tolerance

Among the numerous phytohormones involved in diverse physiological pathways underlying abiotic stress response, ethylene plays a key role in drought and heat response [30,75]. Ethylene is a gaseous hormone involved in signal transduction, playing vital roles in cell growth, seed germination, senescence, abscission, budding, fruit ripening, and stress response [76,]. Indeed, ethylene serves key functions in the regulation of various plant growth mechanisms by mitigating severe damage. High salinity or drought under ABA suppression activates the expression of ERFs. Transgenic *Arabidopsis* plants showed increased tolerance of salinity, drought, and heat stress. The overexpression of ERF-like transcription factors has been implicated in stress tolerance in various plants, such as tomato [18], tobacco, and *Arabidopsis thaliana* [66,68]. Abiotic stress-related genes regulating various underlying molecular and cellular activities retain a biological stigma, and such genes can be effectively targeted with the CRISPR–Cas9 genome editing technique. To date, only a few studies have demonstrated the role of genetic engineering for abiotic stress in developing crops resilient to climate change. ERFs are transcription factors that play critical roles in plant signaling cascades, being involved in diverse stress-responsive mechanisms. ERFs are uniquely diverse plant transcription factors that have been explored in studies evaluating the precise mechanisms of stress response in crops [55].

In maize, novel variants of ARGOS8, a negative regulator of ethylene response, were produced using the CRISPR–Cas9 system, and this variant was more drought tolerant that the wild type. The CRISPR-edited lines showed increased grain yield under the field conditions during the dry season. In wheat protoplast, the CRISPR–Cas9 system was effective in the targeted alteration of the stress-responsive transcription factors wheat ethylene-responsive factor 3 (*TaERF3*) and wheat dehydration-responsive factor binding protein 2 (*TaDREB2*) (Figure 2, Table 1). In rice, RNA interference-mediated knockout of *OsERF109* greatly increased drought tolerance [77]. Similarly, the CRISPR–Cas9 system targeting *OsERF109, OsBIERF4, OsBIERF3*, and *OsBIERF1*, which belong to the ERF (Ethylene responsive factors) family, could improve abiotic stress tolerance in rice. Therefore, genome editing techniques can be used to enhance tolerance of several abiotic stresses. CRISPR/Cpf1, an emerging base editor, appears to be highly effective and reliable in accelerating the development of abiotic stress-tolerant rice cultivars [18,32].

## Multigenic stress tolerance in crops

Abiotic stress is one of the most serious limiting factors in global agriculture, and it is likely to escalate further in the face of climate change. Abiotic stress response a dynamic quantitative trait regulated by several genes and is therefore difficult to handle [50,78]. In this context, the CRISPR–Cas9 method relies on simple DNA/RNA hybrids that confer sequence specificity and can modify almost any sequence in the genome to reveal its purpose [79, 79]. Thanks to its high efficiency, simple design and operation, and ability to simultaneously engineer multiple genomic loci, CRISPR–Cas9 is now preferred over other conventional genome editing tools [80]. The CRISPR–Cas9 technology has two key benefits. First, many sgRNAs can function concurrently with the same Cas9 protein at various loci and second, the specificity for the target DNA can be rapidly modified by programming the sgRNA sequence [55]. Currently, CRISPR–Cas9 is widely used for plant genome editing targeted at particular genes. CRISPR-P is a novel web-based platform for the configuration of sgRNAs in over 20 plant species [81]. In addition, numerous vectors and toolkits have been established for CRISPR–Cas9-based plant genome editing [82]. The application of CRISPR–Cas9 for genetic modification, transcriptional regulation, development of stress-resistant crops, and elucidation of the molecular basis of multigenic stress response is supported by the accessibility of the above evidence [83]. The recently introduced type 2 CRISPR–Cas9 method has further advantages, and this versatile technique has enabled site-specific mutagenesis in a wide range of organisms, particularly model crops. Certain genes of the ERF family, which is associated with fruit ripening and seed extrapolation, show favorable responses to heat, drought, and salinity stress. In rice, the CRISPR–Cas9 method targeted at three negative regulators abiotic stress, namely *OsDERF1, OsERF922*, and *OsRMC*, could produce stable lines with enhanced salinity, drought, and oxidative stress tolerance [74].

## Enhancement of fruit consistency using CRISPR

In tomato fruit, the number of locules originating from flower carpels has the strongest effect on fruit size, explaining 50% of mean variation in fruit size. Locule volume is regulated by several QTLs [84], some of which have been identified. CRISPR–Cas9 rapidly produced bigger tomato fruits by breaking the classic CLAVATA-WUSCHEL (CLV-WUS) stem cell circuit under stress conditions [85]. Eight sgRNAs were engineered to access the promoter region of the *CLV3* gene, and the corresponding transgenic plants developed more fruits and tissues than the wild-type plants. In tomato, a known QTL associated with fruit size and locule number was reconstructed to produce large fruits with numerous locules [86]. For consumers, fruit texture and color are the key components to identify fresh tomatoes [87]. American and European consumers prefer red tomatoes, while Asia consumers prefer pink tomatoes [88,89]. Yellow, pink, and purple tomatoes have been developed by targeting anthocyanin 2 (ANT2), MYB transcription factor 12 (MYB12), and phytoene synthase 1 (PSY1) [90–91]. Furthermore, modifying texture properties for long shelf life has long been an issue for scientists. Inhibition of a ripening inhibitor or DNA demethylase 2 using CRISPR can produce incompletely ripe fruits with long shelf life [92,93]. Nevertheless, these fruits typically do not produce extreme color, resulting in bad taste and low nutritive values. It is important to procure fruits with a reasonable shelf life without impacting other consistency traits. Two research groups reported effective fruit softening by silencing pecta lyase (PL) and alcobaca (ALC) without decreasing the organoleptic and nutritional content of tomatoes [94,95], indicating that the CRISPR method may be an exemplary tool for improving fruit crops.

## Major applications of CRISPER–Cas9 in crops and other fields

At present, CRISPR–Cas-based genome editing is extensively used in nearly all agronomically important crops, including cotton, maize, rice, wheat, soybean, and potato, as well as biofuel crops, including switchgrass. Sugar is an essential part of daily lives as well as biofuel production. Sugarcane and sugar beet are the only two crops that generate substantial levels of sugar. In rice, [96], used CRISPR to knock out GCS1, resulting in fertilization failure and pollen tube-dependent ovule enlargement morphology (POEM). Interestingly, the POEMed-like rice ovule (‘endosperm-focused’) could develop to near-normal sized seed, contrary to previous observations in *Arabidopsis* in which the gcs1 ovules (‘embryo-focused’) were aborted relatively early. The POEMed-like rice ovules contained 10–20% sugar, with a very high sucrose concentration (98% of all sugars) [96]. Transcriptomic studies indicated that in osgcs1 ovules, starch biosynthetic genes, which convert sucrose to starch, were downregulated. Overall, [96], findings indicate that pollen tube content release is sufficient to cause sucrose unloading in rice ovules. Therefore, effective fertilization is required to initiate sucrose–starch conversion [96]. These observations may pave the way for the development of new sugar-producing crops suitable for cultivation in diverse climatic areas.

Foods with high amylose and resistant starch (RS) content can significantly enhance human health and reduce the risk of major noninfectious diseases. Wheat (*Triticum aestivum L*.) is an important staple food crop worldwide. Nevertheless, the grain RS content of modern wheat cultivars is low. [97] developed high-amylose wheat using CRISPR–Cas9-mediated targeted mutagenesis of *TaSBEIIa* in the contemporary winter wheat cultivar ‘Zhengmai 7698ʹ (ZM) and the spring wheat cultivar ‘Bobwhite’. The authors produced various transgene-free mutant lines of ZM and Bobwhite with partial or triple-null *TasbeIIa* alleles. Analysis of starch composition, structure, and characteristics revealed that the effects of partial or triple-null alleles were dose-dependent, with the triple-null lines exhibiting more significant effects on starch content, fine amylopectin structures, and physicochemical characteristics [98]. The flour made from the grains of the triple-null lines had considerably higher levels of amylose, RS, protein, and soluble pentosan, all of which are beneficial to human health. Analysis of baking quality demonstrated that the high-amylose flours may be utilized as additives or to make cookies [98]. Overall, through targeted mutagenesis of TaSBEIIa using CRISPR–Cas9 in both winter and spring wheat cultivars, [97], successfully modified starch content, structure, and characteristics to produce transgene-free high-amylose wheat [99–101].

*Camelina sativa* has emerged as a promising low-input oilseed crop. *Camelina* oil must be improved for optimum fatty acid content, which can fulfill the demands for various uses. Camelina seed contains high amounts of C20-C24 very-long-chain fatty acids (VLCFAs), which are undesirable. [102], demonstrated that these VLCFAs can be efficiently reduced by deactivating fatty acid elongase1 (FAE 1) in Camelina. Allohexaploid Camelina harbors three different alleles of the FAE1 genes. A mutation in the FAE1-B gene induced using ethyl methanesulfonate (EMS) resulted in 60% decrease in VLCFA content of the seed. Homozygous knockout mutants were successfully generated in a single generation by simultaneously targeting the three FAE1 alleles using the CRISPR technology with egg cell-specific Cas9 expression. VLCFA content in the mutants dropped to <2% of the total fatty acid content, compared with approximately 22% in the wild type; however, the levels of C18 unsaturated fatty acids were enhanced. In terms of seed physiology and plant development, the fae1 mutants were indistinguishable from the wild type. Further, FAE1 knockout increased oleic acid or α-linolenic acid content in *Camelina* oil, which are beneficial for industrial or food/feed purposes [102].

Certain fruits, such as tomato and peach, have long been a source of contention in terms of storage and shipping. When fruits are fully grown and have good flavor, they become soft and are difficult to store for a long time and transport over long distances. [95], employed CRISPR–Cas-based genome editing to achieve both ALC mutagenesis and replacement in tomatoes. Similar to that in other plant species, CRISPR HDR-mediated gene substitution in tomato proved far more challenging than CRISPR–Cas-mediated knockout mutagenesis [95]. CRISPR-based genome editing enhanced the storage performance and shelf life of tomato without affecting other agronomic parameters, such as plant growth and fruit firmness [95]. [103], developed tomato plants with altered fruit ripening using a CRISPR–Cas9-mediated knockout of tomato ripening-related lnRNA1459 [101].

As the initial stages of gene repair, current genome editing methods introduce one to two double-stranded DNA breaks at target loci [77]. Although point mutations cause majority of the known genetic disorders, existing techniques to repair point mutations are ineffective, resulting in excess random insertions and deletions (indels) at the target loci due to cellular response to double-stranded DNA breaks [77].

The discovery of ‘base editing,’ a novel method of genome editing that allows for the direct and irreversible conversion of a target DNA base to another in a programmed manner without the need for the fragmentation of the double-stranded DNA backbone or the use of a donor template. [77], developed CRISPR–Cas9 fusions using cytidine deaminase, which can be programmed using a guide RNA do not induce double-stranded DNA breaks while facilitating the direct conversion of cytidine to uridine, resulting in a C →T (or G → A) substitution. The resultant ‘base editors’ convert all cytidine bases within a five-nucleotide window and can quickly repair a wide range of point mutations relevant to human disorders. Second- and third-generation base editors that fuse a uracil glycosylase inhibitor and use a Cas9 nickase targeting the non-edited strand could modify the cellular DNA repair response to favor desired base-editing consequences in four transformed human and murine cell lines, resulting in permanent correction of 15–75% of total cellular DNA with minimal (typically 1%) indel formation. Therefore, base editing has widened the scope and efficiency of point mutation-based genome editing [98,104]. Most genetic variants that cause disease 1 are difficult to repair effectively without generating additional byproducts [98].

Prime editing a dynamic and concise genome editing method that uses a catalytically impaired Cas9 endonuclease fused to an engineered reverse transcriptase program with a prime editing guide RNA (pegRNA), which both specifies the target site and encodes the desired edit to directly write new genetic information at a specified site in DNA. [105], conducted over 175 modifications in human cells, including targeted insertions, deletions, and all 12 types of point mutations, without the introduction of double-strand DNA breaks or the use of donor DNA templates.

[105], used prime editing in human cells to correct the primary genetic causes of sickle cell disease (requiring a transversion in HBB) and Tay–Sachs disease (requiring a deletion in HEXA) as well as to install a protective transversion in PRNP and precisely insert various tags and epitopes at the target loci. With different degrees of efficiency, prime editing was successful in modifying the four human cell lines and primary post-mitotic mouse cortical neurons. Prime editing is more efficient and produces fewer byproducts than homology-directed repair, and it has complementary strengths and weaknesses compared to base editing. As such, prime editing generated considerably less off-target editing than Cas9 nuclease-based editing at a known Cas9 off-target site. Therefore, prime editing has significantly widened the scope and ability of genome editing and, in principle, has the potential to repair up to 89% of known genetic variations related to human disorders [105].

Overall, since its introduction as a genome editing technology, CRISPR–Cas has garnered much attention from the scientific community and commercial sector for treating human genetic disorders. Over the past decade, significant progress has been made in the application of CRISPR–Cas genome editing technology in the clinical and preclinical contexts to cure human genetic abnormalities, screen and diagnose human disorders, and conduct basic biomedical research.

## MicroRNAs (miRNAs)

MicroRNAs (miRNAs) are a diverse family of endogenous, small RNA molecules that regulate the expression of genes involved in various developmental processes and signaling pathways [106, 6]. Recent studies have demonstrated that drought stress leads to aberrant expression of several miRNAs, implying that these molecules can be used as novel targets for genetic improvement of plant resistance to/tolerance of specific stresses. Furthermore, miRNAs respond to drought stress in miRNA-, stress-, tissue-, and genotype-dependent manners. Under drought stress, miRNAs function by modulating target genes within the miRNA–target gene network and affecting signaling pathways and growth. Drought-induced miRNAs down regulate the negative regulator of drought tolerance, whereas drought-inhibited miRNAs promote the accumulation and function of positive regulators. Drought treatment induced miR168 and miR396 expression in *Arabidopsis* [6, 107] and tobacco [108] but inhibited it in rice. Further, drought treatment down-regulated miR408 expression in rice, peach, and cotton [109] but up regulated it in *Arabidopsis* [107], *Medicago*, and barley [109]. Majority of the miRNA-based research was focused on identifying miRNAs that are sensitive to various stresses and analyzing the variations in their expression profiles throughout the treatment, primarily using deep sequencing and other expression analyses, such as quantitative real-time PCR studies. Deep sequencing was used to identify 17 drought-specific miRNAs in switchgrass, 4 of which are conserved and 13 are switchgrass-specific [110]. [111], discovered 21 miRNA gene families in *Populus trichocarpa*, including 48 miRNA sequences, only 11 of which are conserved in *P. trichocarpa* and *Arabidopsis*. Additional functional and expression analyses are warranted in the future to elucidate common miRNA-mediated regulatory mechanisms underlying drought tolerance. The ideal approaches to identify the precise functions of different miRNAs in response to environmental stresses include the use of artificial miRNAs and overexpression or knockout/down of the miRNAs and their targets.

## Conclusion and future prospects

Drought, which negatively affects plant growth and production, is a critical form of environmental stress. Drought stress affects diverse biochemical aspects, in addition to morphological and physiological parameters, which are essential for plant growth. In diverse climatic regions, soil water scarcity and drought can lead to persistent low accessibility of water to plants. In addition, uncertain and spontaneous climatic variations throughout the plant growth cycle can worsen the effects of water deficit. With rising water shortage and rapid climate change, the impacts of drought have become more severe. In this light, significant advances in genomic approaches have opened new windows for deciphering of mechanisms of plant response to drought stress. Specifically, CRISPR–Cas-mediated genome editing has proven a dynamic tool for rapid and high-throughput reconfiguration of endogenous genes. However, relative to the percentage of reviews on different plants, only a few studies have explored the application of CRISPR–Cas-mediated genome editing to improve crop tolerance of drought stress (Figure 2, Table 1). The bottlenecks for genome-edited crops are the discovery of target genes, effective delivery of CRISPR machinery to the right cells and regeneration of various crops. Particular attention to drought stress response genes and drought stress-induced transcriptional networks is required to address the issue of target discovery. In addition, comparative genome-wide analysis will provide a solid foundation for further discovery of the potential target genes in crops.

Over the past decade, significant progress has been made in the discovery, modification, and application of CRISPR–Cas systems in gene functional analyses, clinical research, and crop improvement studies. By targeting several agronomically relevant gene regulators, CRISPR–Cas could dramatically improve plant tolerance to drought stress and enhance average crop production. In plants, CRISPR–Cas-based genome editing closely depends on plant tissue culture-based gene transformation. However, modifying the plant growth medium and culture conditions, such as starvation and drought treatment, could substantially enhanced the plant regeneration capability, and regenerated plants. In addition, comparative genome-wide assessments can provide a factual basis for the exploration of additional potential target genes in plants. As CRISPR–Cas-mediated plant genome editing still faces many challenges, deciphering regulatory mechanisms underlying drought stress tolerance in different plants using genomic strategies will aid the application of this system in various crops.

## Research highlights

- Climate change impacts agriculture by affecting water distribution and temperature
- Genetic engineering can increase drought tolerance and reduce crop losses
- CRISPR–Cas9 has attracted attention as a potent tool to induce hereditary mutations
- Editing efficiency and target identification of plant CRISPR–Cas9 need improvement
- CRISPR–Cas9 may be fundamental method to ensure global food security under climate change

## Authors’ response

All authors have read and approved the manuscript, and ensure that this is the case.
